# Radical nephrectomy for a giant chromophobe renal cell carcinoma diagnosed > 17 years previously: a case report and literature review

**DOI:** 10.3389/fonc.2024.1352689

**Published:** 2024-04-03

**Authors:** Jianhua Lan, Dong Lan, Wenqiang Yuan, Qiao Ying, Jiahong He, Yonglin Gu

**Affiliations:** ^1^ Department of Urology, People’s Hospital of Guang’an City, Guang’an, Sichuan, China; ^2^ Department of Cardiac and Vascular Surgery, People’s Hospital of Guang’an City, Guang’an, Sichuan, China; ^3^ Department of Cardiology, People’s Hospital of Guang’an City, Guang’an, Sichuan, China

**Keywords:** gigantic renal cancer, radical nephrectomy, chromophobe renal cell carcinoma, kidney cancer, kidney

## Abstract

Early diagnosis of renal cell carcinoma relies on imaging tests such as ultrasound, computed tomography, or magnetic resonance imaging. Since surgery is associated with a favorable prognosis, the standard treatment for clinically limited renal cell carcinoma remains surgical resection. Among asymptomatic patients with localized renal cell carcinoma, a small number refuse surgical treatment and survive. We report a case involving a 59-year-old female who underwent a difficult radical nephrectomy 17 years after being diagnosed with malignant tumors due to primary renal cell carcinoma.

## Introduction

Renal cell carcinoma (RCC) accounts for 5% of malignant tumors in males and 3% in females (1). With the development of medical imaging technology, an increasing number of RCC cases are diagnosed at an early stage. Therefore, in practice tumors with extreme size are rarely encountered unless the patient has no symptoms, does not undergo regular physical examinations, or refuses treatment after diagnosis (2). The main histological types of kidney cancer include clear cell, papillary, chromophobe renal cells, and collecting duct carcinoma. Other rare types of RCCs include renal medullary carcinoma and unclassified RCC (3). Most patients diagnosed with kidney cancer opt for active treatment, while a small minority may choose active surveillance.

In this article, we report the treatment of a patient with a huge renal mass of 5.5 kg that had been diagnosed 17 years previously.

## Case presentation

**Figure 1:**
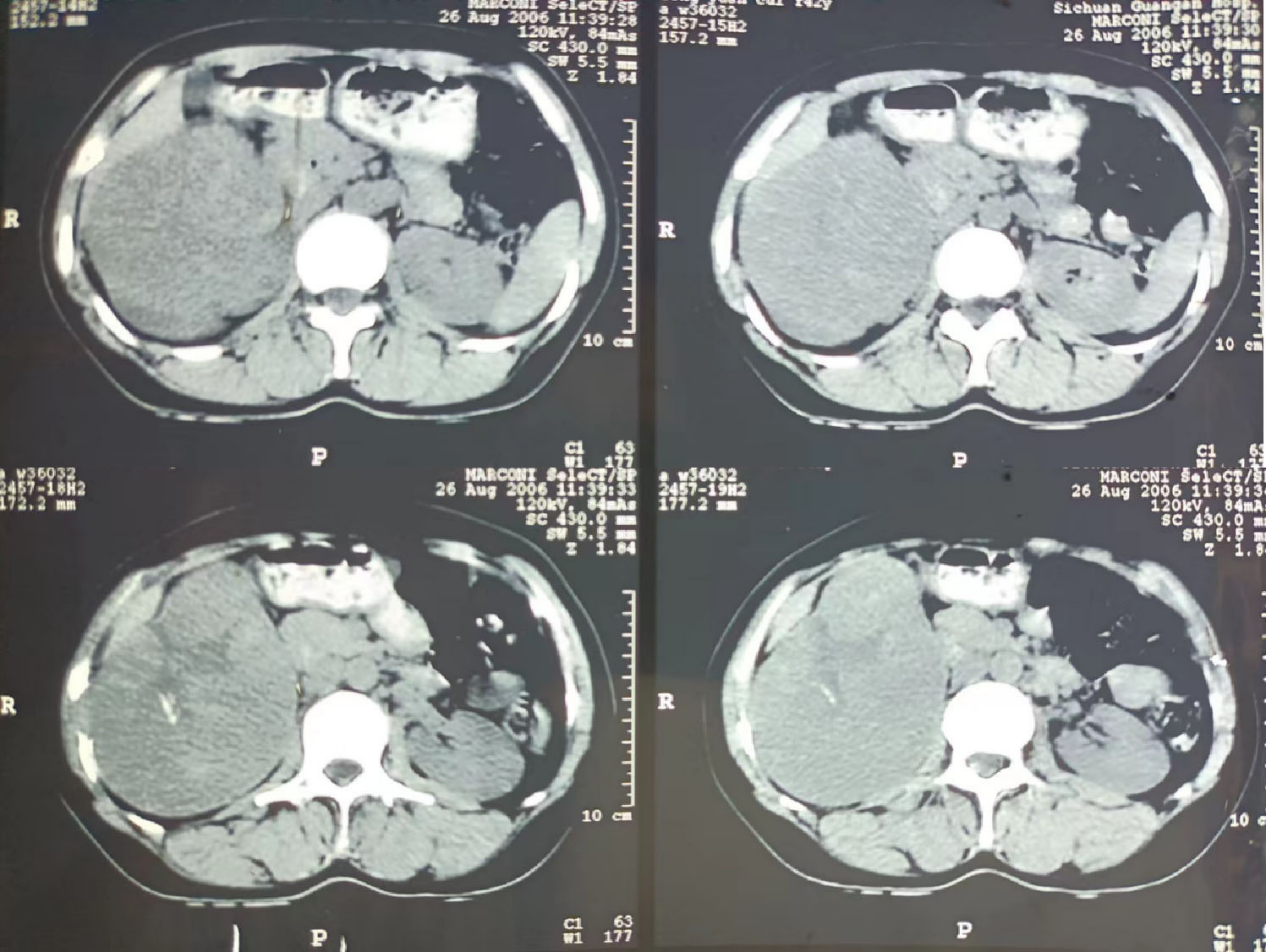
A computed tomography (CT) scan conducted 17 years ago revealing a mass with a maximum cross-sectional diameter of 13 × 8 cm in the right kidney is shown. Several calcifications within the tumor are observable.

The reporting of this study conforms to CARE guidelines (4). A 59-year-old female was referred to our hospital with a large abdominal mass and abdominal pain. She reported early satiety, anorexia, weakness, and weight loss within one month of admission. Seventeen years previously, the computed tomography (CT) examination revealed a 13-cm right renal mass without gross hematuria (**Figure 1**). She was advised to undergo surgery; however, owing to financial constraints, she refused.

**Figure 2:**
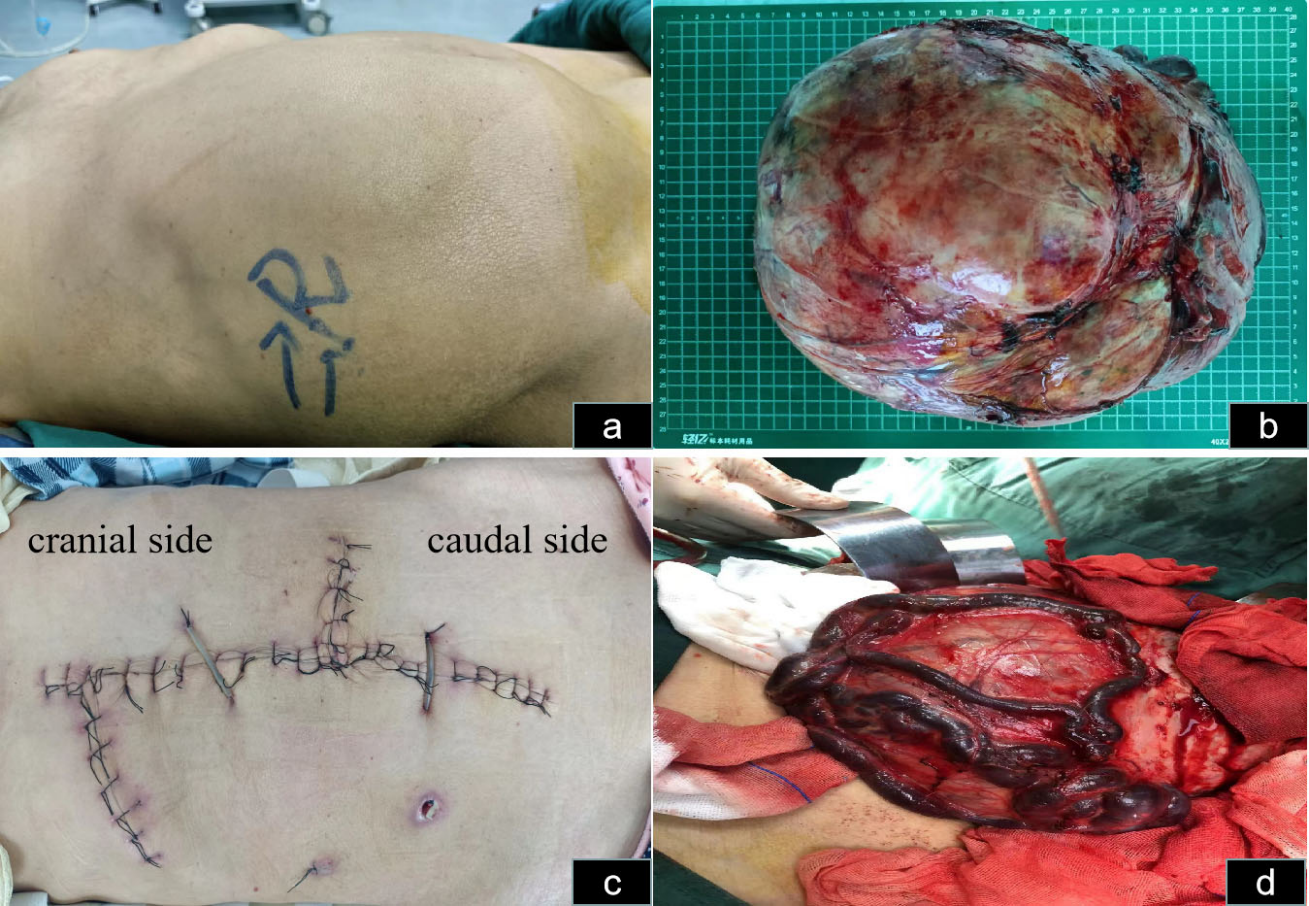
Abdominal incision and tumor status *in vivo* and *in vitro*. **(A)** Abdominal distension, mainly on the right side in the supine position, is observable. **(B)** The hypervascularized tumor after excision is shown. **(C)** The skin incision selected was a midline abdominal incision, along with right upper abdominal oblique and left midline transverse incisions. **(D)** The veins on the surface of the mass are noticeably enlarged and dilated.

**Figure 3:**
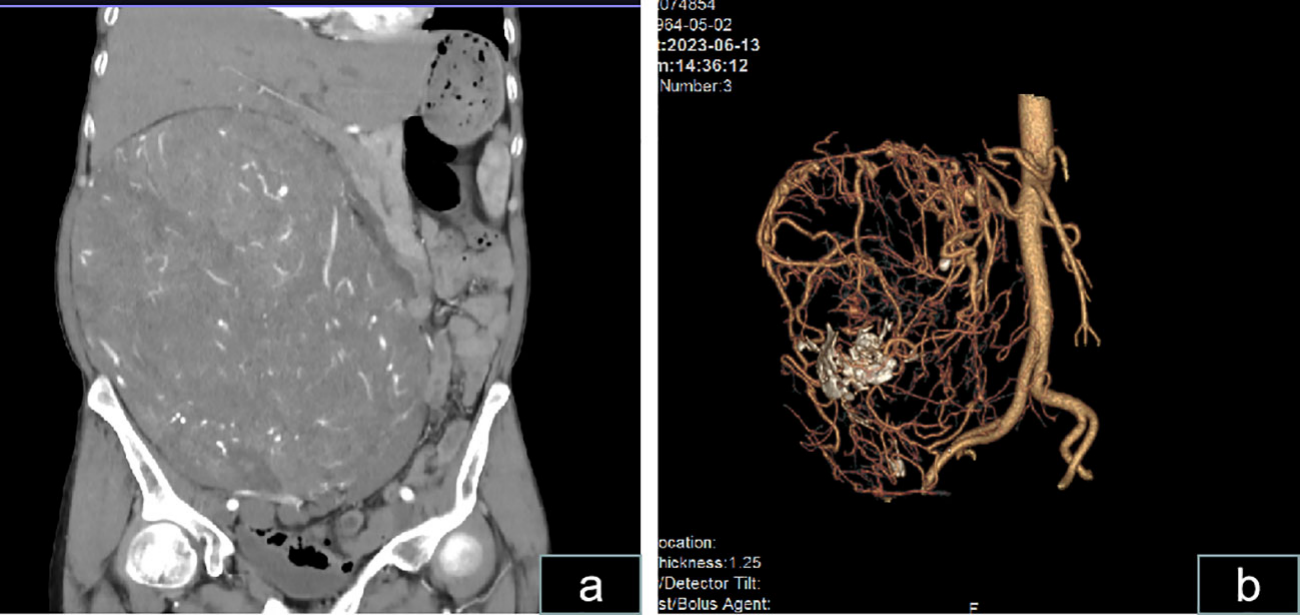
Computed tomography scans show a massive abdominal mass. **(A)** CT scan revealing a massive abdominal mass with dimensions of approximately 25.8 × 22.1 × 15.6 cm exhibiting irregularity and enhancement is shown. **(B)** Vascular reconstruction revealing an extremely rich blood supply for the mass and the presence of a few calcifications within the mass is shown.

Physical examination in the supine position revealed significant abdominal distension, mainly on the right side (**Figure 2A**). The body mass index (BMI) of the patient was 21. On hematological evaluation, hemoglobin (Hgb) was 8.4 g/dl. Other biochemical parameters were normal. Abdominopelvic ultrasonography revealed a large mass measuring 28 × 16 cm. A large hypervascularized mass occupying the right side of the abdomen without evidence of direct inferior vena cava invasion was evident on computed tomography urography (CTU) (**Figure 3**). Chest CT was negative for metastasis. Tumor marker levels (CA19-9 and carcinoembryonic antigen) were within normal ranges.

**Figure 4:**
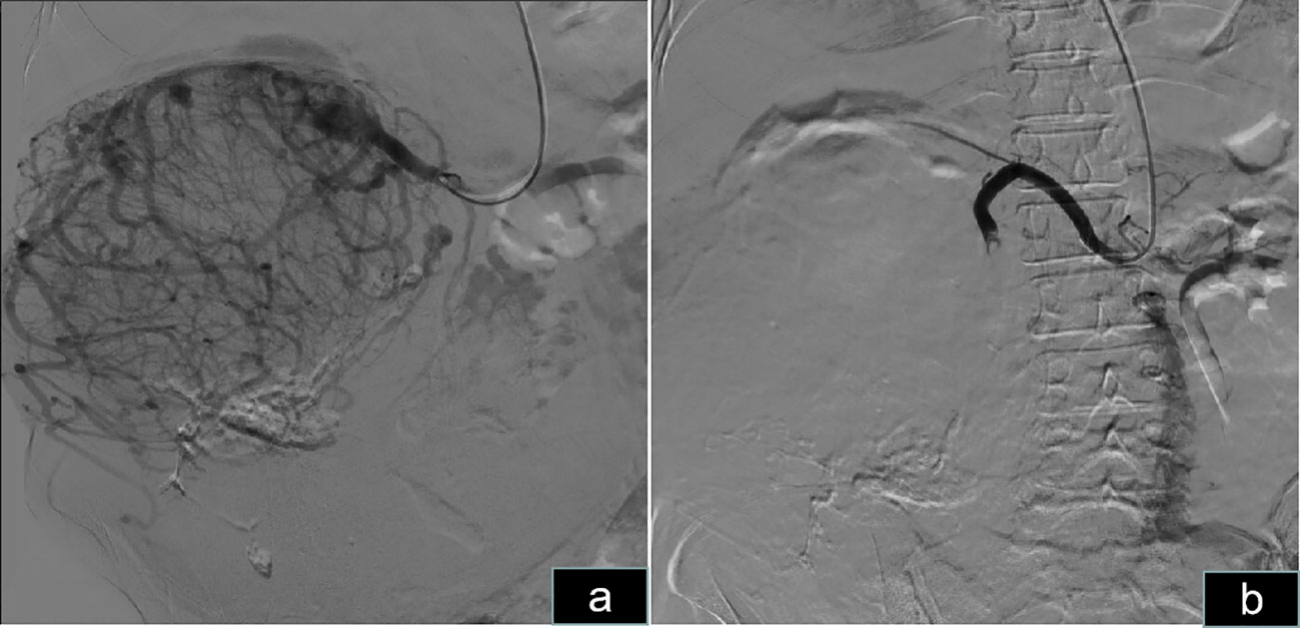
Preoperative right renal artery embolization. **(A)** Angiography of the right kidney revealed abundant blood supply to the right renal tumor. **(B)** Embolization of the right renal artery revealed that the blood supply to the tumor was blocked.

The patient was informed of the radical nephrectomy and other alternative options such as interventional embolization, active monitoring, targeted therapy, etc. The patient strongly requested surgical resection. The patient underwent a right radical nephrectomy via a laparotomy incision. Owing to the extremely large size and abundant blood supply of the tumor, it originated in the right kidney and displaced all intestines to the opposite side. Preoperatively, right renal artery embolization was performed to reduce tumor blood supply and minimize intraoperative bleeding (**Figure 4**). Bowel preparation was performed preoperatively to reduce complications related to intestinal injury. Due to the massive size of the tumor, midline abdominal, right upper abdominal oblique, and left midline transverse incisions were made (**Figure 2C**). Abdominal cavity exploration revealed tumor surface veins were significantly dilated and tortuous, making separation prone to bleeding (**Figure 2D**). After opening the outer side of the ascending colon, gradual separation of the ascending colon and duodenum from the tumor was performed. Subsequently, an inferior incision and separation of the tumor were performed, followed by ligation and division of the right ureter and right ovarian vein. Gradual dissection of the tumor from the surrounding tissues was performed upward and inward. Preoperative renal artery embolization was performed preoperatively, and ligation and division of the right renal vein were performed during surgery, followed by ligation and division of the right renal artery. Finally, the tumor was excised from the liver. Owing to the tight adhesion between the right adrenal gland and the tumor, a complete resection was performed.

The estimated intraoperative blood loss was 1,200 mL, with six units of blood transfused. Postoperatively, the tumor weighed 5.5 kilograms (**Figure 2B**). The mass sections were grayish yellow and grayish brown and were firm and medium in texture. Focal congestive bleeding was observed, and no obvious necrosis or vascular or lymphatic vessel infiltration was observed. Pathological examination indicated chromophobe RCC, and immunohistochemistry revealed weakly positive RCC expression, focally positive expression of PAX-8, and positive expression of CK7, CD10, and CD117. Downregulation of CK20, VIM, P504s, CATX, and HMB45 levels were observed. Ki67 accounted for only 2% of cells. The patient was followed up for three months, throughout which time no recurrence was detected. Adjuvant therapy was not administered.

## Discussion

Here, we report the use of radical nephrectomy via a laparotomy incision to treat a patient with a giant RCC tumor. Currently, there is no clear definition of gigantic cell carcinoma in terms of size and tumor characteristics. Takeda et al. (5) reported the world’s largest renal tumor, measuring 43 cm and weighing 13 kg. The pathological type of the tumor was identified as type 1 papillary RCC. There are several reasons why kidney cancers grow into large masses. Our patient did not undergo regular health checkups for the urinary system. Further, RCC tends to produce erythropoietin and can generate new blood vessels to support its growth (6). In our patient, CTU and intraoperative findings confirmed that the tumor had a rich blood supply. Furthermore, the absence of symptoms and knowledge of a pathological type also contribute to the formation of a large mass in renal cancer. In this patient, the pathological results cannot be determined due to the absence of biopsy 17 years ago. However, no local invasion or metastasis was found in the 13 centimeter sized kidney tumor at that time, indicating good tumor differentiation and low malignancy.

The relationship between giant renal cancer and sex remains unclear. However, case reports indicate that males are more prone to developing giant masses than females (5, 7–10). Our patient underwent surgical treatment > 17 years after her tumor was discovered primarily because she had been asymptomatic. To the best of our knowledge, this is the longest reported survival time in a patient with kidney cancer who did not receive any treatment. Moslemi et al. (11) reported a patient who underwent surgery nine years after their initial malignant kidney tumor diagnosis. Interestingly, the pathological results of Moslemi and our current patient differed, with those of the prior case indicating clear cell carcinoma and the current pathological result suggesting chromophobe carcinoma.

Most patients with renal cancer undergo immediate surgical treatment; therefore, the natural progression of renal masses has not been adequately studied. Historically, RCCs have had slow growth rates of approximately 1–10 mm/year (12). The growth rate of tumors may be influenced by multiple factors including tumor size, number and type of genetic mutations, blood supply, and host immune status. Studies conducted by Ameri et al. (13) revealed differences in the linear tumor growth rates before and after six months. No correlation was shown between tumor linear growth rate and the Fuhrman grading system, gender, histology, or age (13). The study conducted by Finelli et al. (14) showed that the growth rate of renal cell carcinoma subtypes varies, and the growth rate of clear cell RCC was faster than that of papillary type 1, which may reflected the individual host and tumor biology. However, these studies are all active monitoring studies targeting small renal masses, and there are currently no reports on the natural course of large renal masses. In our patient, the tumor grew approximately 0.89 cm per year after diagnosis.

Surgical intervention is the optimal treatment for localized renal tumors, whereas smaller tumors may be treated with radiofrequency ablation and cryotherapy. Giant renal tumors require open surgery because they occupy a significant portion of the abdominal space. The procedure should be performed by an experienced team including urologists, interventional physicians, vascular surgeons, gastrointestinal surgeons, and anesthesiologists. Preoperative CTU examination can provide information about the nature of the mass, the patient’s blood supply status, and the relationship between the tumor’s surrounding organs, aiding in surgical planning. Because the renal vein is located anterior to the renal artery, ligating the renal artery during open surgery for giant renal tumors can be challenging. Preoperative embolization of the renal artery is an alternative approach. There is currently controversy over the surgical timing after arterial embolism. Schwartz et al. (15) believed that the optimal surgical timing was 24-48 hours after embolization, while Çelebioğlu et al. (16) believed that surgical intervention within 24 hours after embolization was better. The timing of renal artery embolization is crucial, as premature embolization may lead to post-embolism syndrome and perirenal edema, which increases bleeding during surgery. We chose immediate embolization before surgery without any evidence of peritumoral edema. During embolization, we prioritized ligation of the renal vein, followed by ligation of the renal artery. Adequate bowel preparation before surgery is essential, and if intestinal injury occurs, prompt repair should be performed.

The fact that the tumor coexisted in our patient for more than 17 years without distant metastasis may be related to multiple factors. Several large studies have indicated that the prognosis of chromophobe RCC is much better than that of clear cell and papillary RCC. Chromophobic renal cell carcinoma and oncocytoma are two subtypes of renal tumors with strikingly similar anatomical origins and histological features, and their accurate differentiation is a significant challenge. CT, MRI and other imaging examinations are helpful for differentiation, but final diagnosis requires histopathology (17, 18). Most chromophobe RCCs have a favorable prognosis and a low risk of metastasis. Nonetheless, there is evidence suggesting that chromophobe RCCs are prone to metastasize to the liver (19). High expression levels of CK7 and CD117, and low expression levels of VIM are considered characteristics of chromophobe RCC (20). Ki-67 accounts for only 2% of cells, indicating low tumor cell proliferation activity, which may be a reason why our patient’s tumor grew significantly over time without distant metastasis. A multi-institution study conducted by Ohashi et al. (21) concluded that age and T stage were the main independent factors that predicted survival in patients with chromophobe RCC. Our patient was followed up for six months. No recurrence or metastasis were observed. Long-term follow-up will be continued in the future.

## Conclusion

This is the first report of massive renal tumor observation for 17 years without evidence of metastasis. A minority of renal tumors can grow into large masses owing to their rich blood supply and lack of obvious symptoms. The data from this case report support the observation that even larger cell carcinomas can be managed through surveillance and that delayed intervention does not appear to have adverse effects.

## Data availability statement

The original contributions presented in the study are included in the article/supplementary material. Further inquiries can be directed to the corresponding author.

## Ethics statement

Written informed consent was obtained from the individual(s) for the publication of any potentially identifiable images or data included in this article.

## Author contributions

JL: Data curation, Funding acquisition, Writing – original draft, Writing – review & editing. DL: Data curation, Formal analysis, Investigation, Writing – original draft. WY: Data curation, Writing – original draft. QY: Investigation, Resources, Data curation, Writing – original draft. JH: Data curation, Formal analysis, Investigation, Writing – original draft. YG: Data curation, Formal analysis, Investigation, Writing – original draft.
